# Sequestered Lumbar Disc Mimicking Psoas Abscess: A Case Report

**DOI:** 10.7759/cureus.79781

**Published:** 2025-02-27

**Authors:** Shu Suzuki, Kazuya Okita, Kazuki Abe, Mizuka Suzuki, Yasunobu Takaki

**Affiliations:** 1 Diagnostic Radiology, Tokyo Metropolitan Cancer and Infectious Diseases Center Komagome Hospital, Tokyo, JPN; 2 Orthopedics, Tokyo Metropolitan Cancer and Infectious Diseases Center Komagome Hospital, Tokyo, JPN

**Keywords:** degenerative disc disease, lumbar disc herniation, psoas abscess, sequestered lumbar disc, vertebral spondylodiscitis

## Abstract

We present a rare case of a sequestered lumbar disc herniation mimicking a psoas abscess. A 68-year-old male with a history of tongue cancer surgery had a ring-enhancing lesion in the left psoas muscle at the L2/3 level on contrast-enhanced computed tomography (CT), raising concerns about psoas abscesses related to vertebral spondylodiscitis. Magnetic resonance imaging (MRI) revealed a well-defined mass with low signal intensity on T1-weighted images, high signal intensity on T2-weighted images, and surrounding muscle edema. CT-guided biopsy revealed degenerative intervertebral disc tissue without malignancy or acute inflammation, confirming a sequestered disc herniation as the cause. Sequestered disc herniation is a rare condition in which the disc material migrates away from its origin, presenting similarly to infections or tumors. Imaging features such as ring enhancement in contrast studies can complicate the diagnosis. This case highlights the importance of considering sequestered disc herniation in the differential diagnosis of psoas muscle masses. Further research is needed to better understand the pathophysiology and management of sequestered disc herniation in the psoas muscle.

## Introduction

Lumbar disc herniation is a common medical condition typically observed in individuals in their 30s to 50s. It most frequently occurs in the lower lumbar spine, particularly at the L4/5 or L5/S1 levels [1]. More than 90% of lumbar disc herniations are located within the spinal canal [2]. A sequestered disc herniation, also known as a sequestered disc fragment, a rare form in which the disc material migrates away from its origin, can mimic conditions such as psoas abscesses or spinal tumors on imaging, potentially leading to misdiagnosis [3,4]. While both conditions cause back pain and mobility issues, psoas abscess is an infectious process with systemic symptoms, whereas a sequestered lumbar disc results from disc herniation causing nerve compression. Computed tomography (CT) and magnetic resonance imaging (MRI) are valuable diagnostic tools; however, the overlapping imaging features of sequestered discs and other psoas lesions necessitate careful interpretation. Treatment strategies depend on symptom severity and neurological findings. Conservative treatment is typically the first approach, which includes rest, physical therapy, and medications to manage pain and inflammation. Surgical intervention is recommended for severe cases with significant neurological deficits or persistent pain that doesn’t respond to conservative treatment, aiming to remove the sequestered disc fragment and relieve nerve compression. Herein, we present a rare case of a sequestered lumbar disc mimicking a psoas abscess, highlighting the diagnostic challenges and importance of accurate interpretation in comparison with previously reported cases.

## Case presentation

**Figure 1:**
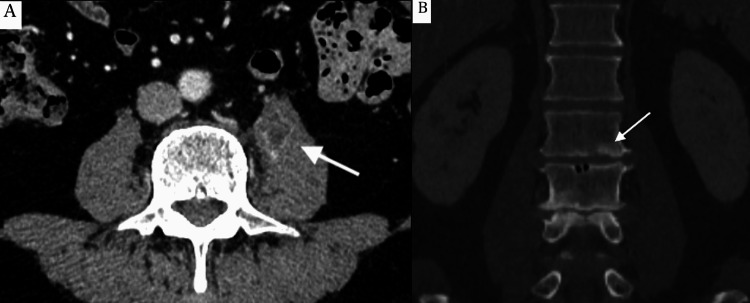
Computed tomography imaging findings (A) A ring-enhancing lesion in the left psoas muscle at the L2/3 level on contrast-enhanced computed tomography (arrow). (B) Deforming changes such as narrowing of the disc space and osteophyte formation were observed. Sclerosis and slight irregularities at the endplates of the L2/3 vertebral bodies were also observed (arrow).

A 68-year-old male patient with a history of tongue cancer surgery in 2017 visited the hospital to search for metastasis as an annual follow-up, and a ring enhancement lesion (28 × 16 × 39 mm) was incidentally detected in the left psoas muscle at the L2/3 level on contrast-enhanced CT. Additionally, deforming changes such as narrowing of the disc space, osteophyte formation, sclerosis, and slight irregularities at the endplates of the L2/3 vertebral bodies were observed, raising concerns about vertebral spondylodiscitis and psoas abscess (Figure 1).

**Figure 2:**
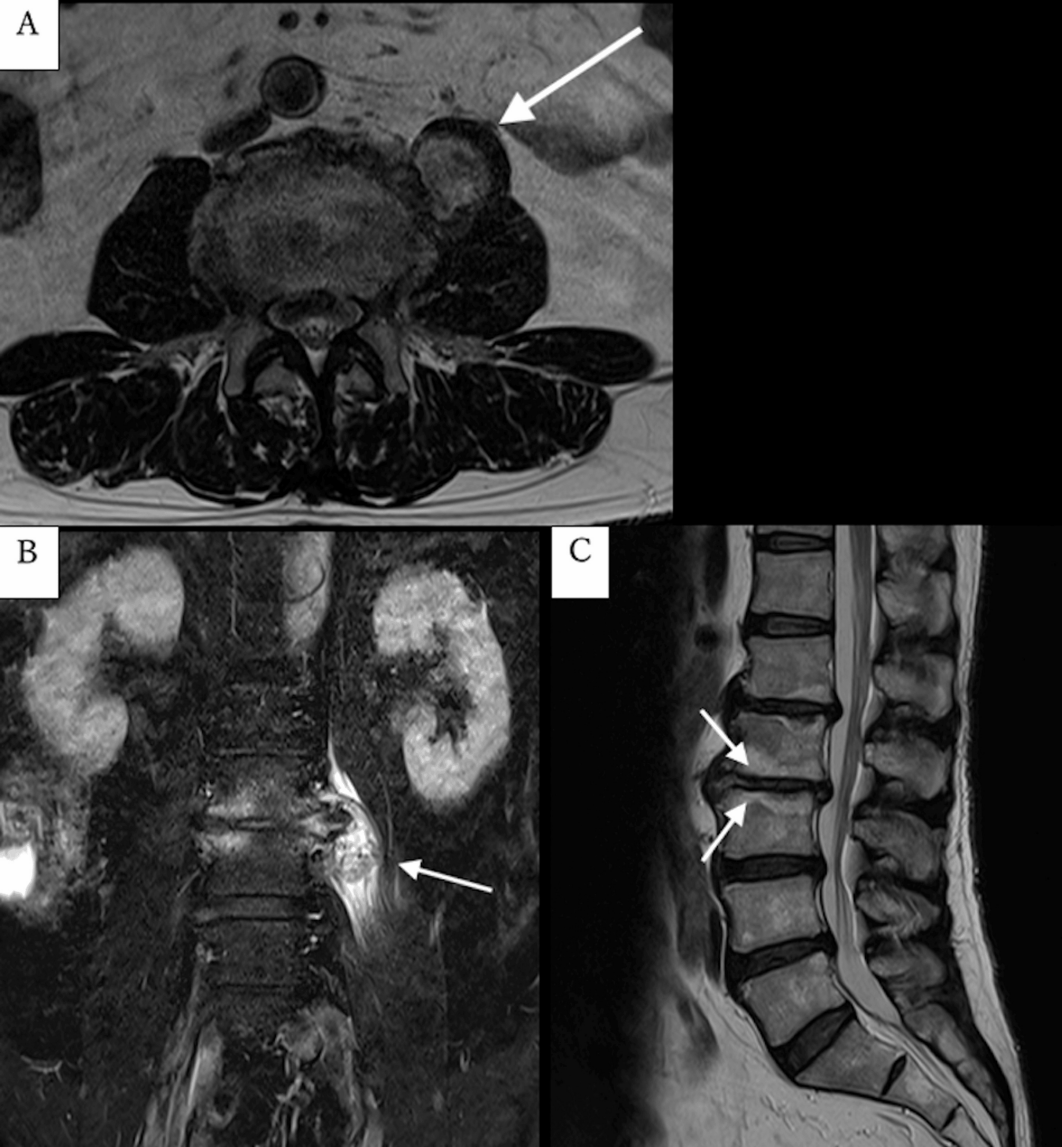
Magnetic resonance imaging findings (A) The left psoas mass shows high signal intensity with mixed low signal on T2-weighted images at the L2/3 level (arrow). (B) The surrounding muscle shows a high signal intensity on short tau inversion recovery (STIR) (arrow). The L2-3 vertebrae adjacent to the mass showed disc bulging, osteophyte formation, and endplate edema. (C) The preserved endplate contour on T1-weighted images at the L2/3 level (arrows).

Other than this lesion, no findings suggestive of tongue cancer recurrence or metastasis were observed. On examination, he was afebrile, walked without pain, and had a normal gait. Laboratory tests, including white blood cell (WBC) and C-reactive protein (CRP) levels, were unremarkable. Blood and urine cultures were negative, and the T-spot test was also negative. An MRI performed two weeks later demonstrated a well-defined mass in the left psoas muscle at the L2/3 level, which showed low signal intensity on T1-weighted images and high signal intensity with mixed low signal on T2-weighted images. The surrounding muscle showed a high signal intensity on short tau inversion recovery (STIR) imaging, suggesting edematous or inflammatory changes. Disc degeneration and osteophyte formation adjacent to the mass were observed at the L2/3 level. Although the high signal intensity at the endplate on STIR raised the possibility of both edematous and infectious conditions, the endplate contour on T1-weighted images was preserved, which was indicative of degenerative changes rather than vertebral spondylodiscitis (Figure 2).

Given the primary concerns regarding a psoas abscess, a CT-guided fine-needle biopsy was performed. CT tomography demonstrated a core biopsy needle in the left psoas muscle. Histopathological examination revealed degenerated intervertebral disc tissue (L2/3) with atrophic skeletal muscle. Although scattered lymphocytic infiltration was observed, there was no evidence of malignancy or acute inflammation. The combination of all the findings was diagnostic of disc sequestration mimicking a psoas abscess. The patient was managed conservatively, and no clinical deterioration was observed during follow-up.

## Discussion

In this study, we present a case of a sequestered lumbar disc mimicking a psoas abscess. The differential diagnosis of a mass in the psoas muscle is broad and includes infection, hematoma, and tumor, with disc herniation being a rare cause [5]. When the extruded disc loses its connection to the parent disc, it becomes a sequestered fragment. As noted in our case, a sequestered disc herniation can mimic a psoas abscess or spinal tumor, causing diagnostic challenges. This presentation has only been reported in a series of 3,000 histologically confirmed disc herniation cases [6] and four case reports [4,7-9]. Four of the five reported cases occurred at the L2/3 level, similar to the present case, and one at the L3/4 level, indicating a predilection for the upper lumbar spine. This is a different trend from previous reports, in which L3/4 and L4/5 levels were more common in free lumbar disc herniation (FLDH), while cephalad involvement of L1/2 or L2/3 was rare [10]. It is unclear why sequestered lumbar discs that mimic psoas abscesses are more common in the upper lumbar spine.

Psoas abscesses can be classified as primary or secondary. Primary psoas abscesses result from the hematogenous spread of occult infection or local trauma, but the etiology remains uncertain. Secondary psoas abscesses typically arise from adjacent infections or pathological conditions, with common causes including vertebral spondylodiscitis, gastrointestinal infections (e.g., appendicitis and diverticulitis), urinary tract infections (e.g., pyelonephritis), and gynecological conditions (e.g., pelvic inflammatory disease). Direct invasion may also occur because of surgical procedures, trauma, or necrotic infections from malignancies [4,11]. While psoas abscess with vertebral spondylodiscitis remained the primary diagnosis on contrast-enhanced CT, MRI findings showed a mass in the psoas muscle with degenerative changes such as disc degeneration, osteophyte formation, and endplate edema at the L2/3 level. Although these changes sometimes overlap with vertebral spondylodiscitis, preservation of the endplate contour on T1-weighted images is indicative of degenerative changes rather than vertebral spondylodiscitis [12]. Repeat imaging can be considered; when the possibility of infection cannot be ruled out, a biopsy should be considered for diagnostic purposes [4].

Sequestered discs typically exhibit low signal intensity on T1-weighted images and high signal intensity on T2-weighted images, which is attributed to inflammation, increased water content, and vascular proliferation [6]. Contrast enhancement often reveals ring enhancement due to surrounding inflammatory cell infiltration [13]. All previous case reports similar to ours have shown ring enhancement on contrast-enhanced MRI [4,6-9]. In our case, although contrast-enhanced MRI was not performed, contrast-enhanced CT revealed ring enhancement of the mass in the left psoas muscle, probably due to a similar mechanism. These findings suggest that ring enhancement is a characteristic feature of sequestered disc herniation; however, it is not specific and should be interpreted with caution because similar imaging features can also be observed in abscesses.

Regarding the management of sequestered disc herniation mimicking a psoas abscess, previous cases have reported that surgical intervention is performed when a clear diagnosis cannot be established through imaging alone or when neurological symptoms are progressive [6-8]. In contrast, two patients were managed conservatively, with one showing complete resolution of the sequestered fragment [4,9]. This phenomenon has also been reported in other studies where large disc herniations, sequestered fragments, and those with ring enhancement may spontaneously regress [14]. In a rabbit model, an increase in fibroblast growth factor (FGF) concentration was shown to be beneficial, as it leads to an increase in disc resorption [15]. As demonstrated in our case, conservative management avoids invasive treatments, thus offering a potential advantage.

## Conclusions

In conclusion, we report a rare case of sequestrated disc fragments mimicking a psoas abscess on imaging. In addition, this report highlights that this rare form of disc herniation is frequently observed at the L2/3 level, although the underlying mechanism remains unclear. Histopathological confirmation prevents unnecessary intervention, and conservative management is an option because spontaneous regression has been reported. When a psoas muscle mass with ring enhancement is observed, sequestrated disc herniation should be considered in the differential diagnosis. Further research is needed to clarify its pathophysiology and identify optimal management strategies.

## References

[1] Läubli R, Brugger R, Pirvu T (2021). Disproportionate vertebral bodies and their impact on lumbar disc herniation. J Clin Med.

[2] Diehn FE, Maus TP, Morris JM (2016). Uncommon manifestations of intervertebral disk pathologic conditions. Radiographics.

[3] Simonin A, Paris O, Brouland JP, Morard M, San Millán D (2018). Degenerative disc disease mimicking spondylodiscitis with bilateral psoas abscesses. World Neurosurg.

[4] Parmar G, Soin P, Sharma P, French C, Han B, Kochar PS (2022). Sequestered disc herniation mimicking psoas abscess: a rare case report. Radiol Case Rep.

[5] Berry AC, Fussell J, Shapira G, Dawson W, Hogue A (2019). Iliopsoas mass, hydroureter, and back squats: using biomechanics and diagnostic persistence to diagnose a germ cell tumor. Ochsner J.

[6] Carvi y Nievas MN, Hoellerhage HG (2009). Unusual sequestered disc fragments simulating spinal tumors and other space-occupying lesions. J Neurosurg Spine.

[7] Chen KT (1997). Cytodiagnosis of a herniated disk presenting as a soft tissue mass. A case report. Acta Cytol.

[8] Kachramanoglou C, Farmer SF, Choi D (2012). Sequestered disc fragment mimicking a psoas abscess. Spine J.

[9] Azizi L, Jaber R, Faddoul S (2024). Psoas muscle sequestered disc mimicking an intramuscular abscess: a rare case report. Radiol Case Rep.

[10] Epstein NE (2002). Foraminal and far lateral lumbar disc herniations: surgical alternatives and outcome measures. Spinal Cord.

[11] Tomich EB, Della-Giustina D (2009). Bilateral psoas abscess in the emergency department. West J Emerg Med.

[12] Schwarz-Nemec U, Friedrich KM, Stihsen C (2020). Vertebral bone marrow and endplate assessment on MR imaging for the differentiation of Modic Type 1 endplate changes and infectious spondylodiscitis. J Clin Med.

[13] Kim H, Kwon BS, Park JW (2018). Posterior epidural migration of a lumbar intervertebral disc fragment resembling a spinal tumor: a case report. Ann Rehabil Med.

[14] Hu C, Lin B, Li Z, Chen X, Gao K (2021). Spontaneous regression of a large sequestered lumbar disc herniation: a case report and literature review. J Int Med Res.

[15] Schroeder GD, Guyre CA, Vaccaro AR (2016). The epidemiology and pathophysiology of lumbar disc herniations. Semin Spine Surg.

